# Vasohibins: new transglutaminase-like cysteine proteases possessing a non-canonical Cys-His-Ser catalytic triad

**DOI:** 10.1093/bioinformatics/btv761

**Published:** 2016-01-21

**Authors:** Luis Sanchez-Pulido, Chris P. Ponting

**Affiliations:** MRC Functional Genomics Unit, Department of Physiology, Anatomy and Genetics, University of Oxford, Oxford OX1 3QX, UK

## Abstract

**Summary:** Vasohibin-1 and Vasohibin-2 regulate angiogenesis, tumour growth and metastasis. Their molecular functions, however, were previously unknown, in large part owing to their perceived lack of homology to proteins of known structure and function. To identify their functional amino acids and domains, their molecular activity and their evolutionary history, we undertook an in-depth analysis of Vasohibin sequences. We find that Vasohibin proteins are previously undetected members of the transglutaminase-like cysteine protease superfamily, and all possess a non-canonical Cys-His-Ser catalytic triad. We further propose a calcium-dependent activation mechanism for Vasohibin proteins. These findings can now be used to design constructs for protein structure determination and to develop enzyme inhibitors as angiogenic regulators to treat metastasis and tumour growth.

**Contact:** luis.sanchezpulido@dpag.ox.ac.uk

**Supplementary information:** Supplementary data are available at *Bioinformatics* online.

## 1 Introduction

Tight regulation of angiogenesis contributes to normal physiology, growth and development, but when misregulated leads to, or dramatically affects, pathological conditions, such as ischaemia, wound healing and cancer (Ferrara and Kerbel, 2005). Angiogenesis is a fundamental step in transitioning tumours from benignancy to malignancy. The use of angiogenesis inhibitors in treating cancer has thus received considerable attention over the last four decades (Bergers and Benjamin, 2003).

Vasohibin-1 (VASH1) was initially identified as a vascular endothelial growth factor inducible gene that regulates endothelial cell migration (Watanabe *et al.*, 2004; Sato, 2013; Sato, 2015). Despite lacking a classical secretion signal, human VASH1 protein is released extracellularly, assisted by a small vasohibin-binding protein which is the only known VASH1-interacting protein (Suzuki *et al.*, 2010). Over-expression of a paralogue, Vasohibin-2 (VASH2), is associated with diverse tumours, with major roles to angiogenesis, malignant transformation, and metastasis (Kim *et al.*, 2015; Kitahara *et al.*, 2014; Koyanagi *et al.*, 2013; Shibuya *et al.*, 2006; Takahashi *et al.*, 2012; Tu *et al.*, 2014; Xue *et al.*, 2013). Knockout mouse studies have revealed the contrasting roles of the two paralogues (Ito *et al.*, 2013). VASH1 is expressed in endothelial cells in zones within which angiogenesis is arrested, whilst VASH2 is expressed in infiltrating mononuclear cells at the sprouting front in which angiogenesis is enhanced (Kimura *et al.*, 2009). The molecular activity of neither paralogue is known, in large part due to a perceived lack of homology to proteins of known structure and function.

## 2 Results and discussion

### 2.1 Computational protein sequence analysis

We initiated our analyses by performing a JackHMMER iterative search (Finn *et al.*, 2015) starting from the human VASH1 protein sequence, against the UniRef50 database (Wu *et al.*, 2006). With the exception of fungi, the vasohibin family is widely distributed in eukaryotes: in animals it is represented from humans to placozoans (*Trichoplax adhaerens*), but is absent from nematodes (*Caenorhabditis elegans*) and hexapods (*Drosophila melanogaster*), and in plants homologues are present from green algae to Bryophytes (*Physcomitrella patens*) but are not in vascular plants. We identified an evolutionarily conserved central region in the vasohibin family, thereby reproducing this unusual phyletic distribution reported in Pfam (Family: vasohibin/PF14822) (Punta *et al.*, 2012). Vasohibin homologous sequences were identified in standard databases (UniProt, GenBank and Joint Genome Institute data) and in manually assembled ESTs and FGENESH+-predicted gene models (Solovyev *et al.*, 2006).

**Fig. 1.**
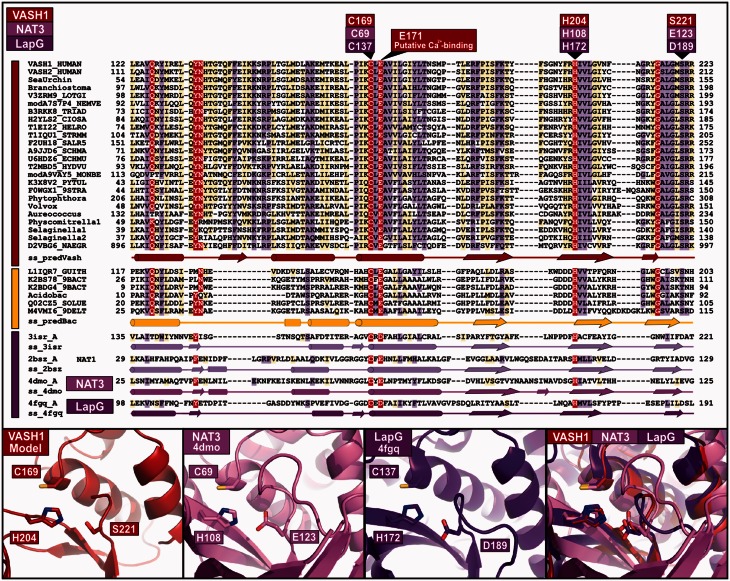
Multiple sequence alignment of representative vasohibin and related families. Multiple sequence alignments for each family were generated with the program T-Coffee (Notredame *et al*., 2000) using default parameters and slightly refined manually. The final superfamily alignment was generated using a combination of profile-to-profile comparisons (Söding *et al*., 2005) and sequence alignments derived from structural super-positions for those families whose tertiary structure is known (PDB IDs: 3isr, 2bsz, 4dmo and 4fgq) (Holm and Sander, 1995). Active site residues are labelled and coloured according to reference protein sequences: VASH1, NAT3 (human arylamine N-acetyltransferase 3), and calcium-dependent periplasmic cysteine protease LapG in red, pink and violet, respectively. A putative calcium-binding residue (human VASH1 E171) is labelled. Families are indicated by coloured bars to the left of the alignment: Vasohibin, Vasohibin-like and transglutaminase-like cysteine protease are indicated in red, yellow and purple, respectively. The limits of the protein sequences included in the alignment are indicated by flanking residue positions. Secondary structure predictions (Jones, 1999) were performed independently for the vasohibin and vasohibin-like families (show in ss_predVash and ss_predBac lanes); these are consistent with X-ray determined secondary structures of the putative cysteine protease from *C. hutchinsonii* (PDB: 3isr) (Stein *et al*., *Midwest Center for Structural Genomics*, unpublished), NAT1 (arylamine N-acetyltransferase-1) from *Mesorhizobium loti* (PDB: 2bsz) (Holton *et al*., 2005), NAT3 (arylamine N-acetyltransferase-3) from *Bacillus cereus* (PDB: 4dmo) (Kubiak *et al*., 2013) and the calcium-dependent periplasmic cysteine protease LapG from *Legionella pneumophila* (PDB: 4fgq) (Chatterjee *et al*., 2012). Alpha-helices and beta-strands are indicated by cylinders and arrows, respectively. The alignment was presented with the program Belvu using a colouring scheme indicating the average BLOSUM62 scores (which are correlated with amino acid conservation) of each alignment column: red (>3), violet (between 3 and 1.5) and light yellow (between 1.5 and 0.5) (Sonnhammer and Hollich, 2005). Sequences are named according to their UniProt identification or common name (details provided in Supplementary Figure S1). Below the alignment are shown a homology-model of human VASH1 and known structures of representative members of the transglutaminase-like cysteine protease superfamily. Catalytic triads are labelled and side chains shown using sticks. The human VASH1 structural model was created using Modeller (Sali and Blundell, 1993). VASH1 model and structures are presented using Pymol (http://www.pymol.org)

Next, we took advantage of HMMer3 and HMMer2 (Eddy, 1996; Finn *et al.*, 2015) to search UniRef50 for more divergent vasohibin homologues using a hidden Markov model (HMM) generated from the central conserved region (corresponding to human VASH1, UniProt: VASH1_HUMAN, amino acids 125–247). With this profile, HMMer3 identified significant (*E*-value = 0.011) sequence similarities between the vasohibin HMM and a cryptophyte algae protein from *Guillardia theta* (UniProt: L1IQR7_GUITH, amino acids 120–227). This *G. theta* protein is a member of an experimentally uncharacterised family (henceforth, the ‘vasohibin-like’ family) drawn from phylogenetically heterogeneous organisms. These include Acidobacteria (*Solibacter usitatus* and *Acidobacteriaceae bacterium*), Proteobacteria (*Bdellovibrio exovorus* and *Reyranella massiliensis*), and eukaryotes (*G. theta, Chaetosphaeridium globosum, Chlorokybus atmophyticus* and the dinoflagellate *Karlodinium veneficum*; Fig. 1 and Supplementary Figure S1).

Profile-versus-sequence (HMMer2 and HMMer3) (Eddy, 1996; Finn *et al.*, 2015) and profile-versus-profile (HHpred) (Söding *et al.*, 2005) similarity searches using an HMM representing the central conserved portion of the vasohibin-like family produced concordant results, identifying robust statistical significance (HMMer2 *E*-value = 0.03 and HMMer3 *E*-value = 1.2 × 10^−^^5^) for the sequence similarity between vasohibin and vasohibin-like families (Supplementary Figure S2).

Unexpectedly, HHpred searches against the PDB70 profile database (Söding *et al.*, 2005), again using the conserved central region of the vasohibin-like family, matched the transglutaminase-like cysteine protease domain of *Cytophaga hutchinsonii* protein (PDB-ID: 3ISR) with a highly significant *E*-value of 6.5 × 10^−^^4^ (Fig. 1 and Supplementary Figure S2). Moreover, in support of this first match, the next most statistically significant matches were to five further members of the transglutaminase-like cysteine protease superfamily [PDB-IDs: 3KD4, 4FGQ (LapG), 2F4M, 2BSZ (NAT1) and 4DMO (NAT3)]. Many members of this superfamily are functionally and structurally characterized enzymes classified in different protein databases, such as: Pfam (Clan Peptidase_CA CL0125, containing 60 families), SCOP (Fold: Cysteine proteinases, subdivided in 22 families) and MEROPS database of peptidases (clan CA, carefully subdivided in 36 families) (Andreeva *et al.*, 2004; Punta *et al.*, 2012; Rawlings *et al.*, 2014).

Additionally, secondary structure predictions of the vasohibin and vasohibin-like families corroborated this proposed homology because they yielded high similarity with known transglutaminase-like cysteine protease superfamily structures (Fig. 1).

### 2.2 Active site conservation, a non-canonical catalytic triad

Proteins of the transglutaminase-like cysteine protease superfamily catalyse various reactions (such as: transglutaminases, proteases, phytochelatin synthases and arylamine N-acetyltransferases) on diverse substrates. Nevertheless, they all contain a conserved catalytic triad (usually Cys-His-Asp/Glu) (Chatterjee *et al.* 2012; Ginalski *et al.*, 2004; Keillor *et al.*, 2014; Makarova *et al.*, 1999, 2000; Polgár and Asbóth, 1986; Vivares *et al.*, 2005). These amino acids are conserved as Cys-His-Ser/Thr in human VASH1 and all its homologues at sites that structurally superimpose onto the transglutaminase active centre residues (Fig. 1). This modified catalytic triad (Cys-His-Ser/Thr) thus appears to be essential for the normal function of the vasohibin family.

Active sites and catalytic mechanisms of transglutaminase-like cysteine proteases have been extensively studied (Keillor *et al.*, 2014; Polgár and Asbóth, 1986). In the classical Cys-His-Asn/Asp catalytic triad, the Cys, activated as a thiolate anion by the His residue, plays the major role in the nucleophilic attack the substrate. Both positions, His and Cys, are completely conserved across all members of the vasohibin family (VASH1 residues C169 and H204) (Fig. 1).

The third position of the catalytic triad (commonly Asn, Asp or Glu) is known to have a secondary role in catalysis by orienting the His side chain to allow the formation of a thiolate/imidazolium ion pair between Cys and His residues (Vernet *et al.*, 1995). Such a role, we suggest, could also be performed by the hydroxyl groups of the conserved Ser or Thr amino acids (VASH1 residue S221) (Fig. 1). This non-canonical catalytic triad (Cys-His-Ser/Thr) present in the Vasohibin family, is not unique to the transglutaminase-like cysteine protease superfamily, because members of the Transglut_core family (Pfam entry: PF01841) (Punta *et al.*, 2012), part of the transglutaminase-like cysteine protease superfamily, also contain a Cys-His-Thr non-canonical catalytic triad (e.g. A0LIH5_SYNFM from *Syntrophobacter fumaroxidans*). Another example of a non-canonical catalytic triad is the Cys-His-(O=C)Trp triad in mSpeB (PDB: 1dki) (Kagawa *et al.*, 2000), where the role of orienting the His residue is taken by the backbone carbonyl group of a Trp amino acid. Despite containing this unconventional catalytic triad, mSpeB is an unusually active protease (Honda-Ogawa *et al.*, 2013).

### 2.3 Activators, inhibitors and regulators in the Transglutaminase-like cysteine protease superfamily

Owing to the higher concentrations of calcium in the extracellular space, diverse secreted proteins possess calcium-dependent activation mechanisms that avoid intracellular activation. Calcium dependent activation is a recurring theme across the transglutaminase-like cysteine protease superfamily (Boyd *et al.*, 2012; Chatterjee *et al.*, 2012; Jang *et al.*, 2014). Calcium-binding sites show a wide range of geometries involving three or four-COOH groups (particularly aspartic and glutamic acids) plus two neutral oxygen donors (usually backbone atoms) (Wang *et al.*, 2009). A calcium-dependent activation mechanism in vasohibin homologues is suggested by the alignment of a conserved amino acid (VASH1-E171) with a known calcium binding residue in LapG protease (LapG-D139) (Fig. 1) (Chatterjee *et al.*, 2012). Experimental evidence will be necessary to confirm these Vasohibin family predicted calcium-binding sites.

Angiogenesis is not a new cellular process for members of the transglutaminase-like cysteine protease superfamily. Tissue transglutaminase (also known as transglutaminase 2), for example, and also different members of the cysteine cathepsin family of peptidases, have been frequently implicated in various aspects of vascular morphogenesis (Turk *et al.*, 2012; Wang *et al.*, 2013). The identification of the Vasohibin family as members of the transglutaminase-like cysteine protease superfamily may further clarify the known relationship between reactive oxygen or nitrogen species (ROS and RNS) and angiogenesis (Huang and Zheng, 2006; Ushio-Fukai, 2007; Ushio-Fukai and Urao, 2009). ROS and RNS are known to inactivate different members of the transglutaminase-like cysteine protease superfamily, such as papain (Xian *et al.*, 2000), arylamine N-acetyltransferase (NAT1) (Dupret *et al.*, 2005) and transglutaminases (Bernassola *et al.*, 1999; Nurminskaya and Belkin, 2012). We thus suggest that the putative catalytic cysteine residues in VASH1 and VASH2 could be substrates of ROS and RNS activity.

Owing to the enzymatic diversity of its transglutaminase-like homologues, our computational analysis is unable to predict the catalytic reaction or substrate of vasohibins. Nevertheless, the discovery of vasohibins as enzyme homologues should now motivate the determination of their protein structures and the development of active site inhibitors that differentiate VASH1 from VASH2. Such inhibitors would be anticipated to modify angiogenesis and thus to be useful for inhibiting metastasis and tumour growth. We note that different inhibitors have already been described for several members of the transglutaminase-like cysteine protease superfamily (Badarau *et al.*, 2013; Chakka *et al.*, 2015; Keillor *et al.*, 2015; Kerr *et al.*, 2009; Kukongviriyapan *et al.*, 2006; Pandey and Dixit, 2012; Sim *et al.*, 2014; Turk *et al.*, 1997; Zhou *et al.*, 2013).

## 3 Conclusion

Given the strong statistical significance of sequence and profile comparisons, and the concordance of secondary structure predictions and conserved active site residues, we have shown that vasohibin proteins are new members of the transglutaminase-like cysteine protease superfamily, and possess a non-canonical Cys-His-Ser/Thr catalytic triad. This insight should immediately help guide experiments to clarify the molecular mechanisms by which VASH1 and VASH2 control angiogenesis.

## Funding

This work was supported by Medical Research Council UK.

*Conflict of Interest:* none declared.

## Supplementary Material

Supplementary Data
