# Supplementary material for: Vasohibins: new transglutaminase-like cysteine proteases possessing a non-canonical Cys-His-Ser catalytic triad
Source: Bioinformatics. 2016 Jan 21;32(10):1441–5. doi: 10.1093/bioinformatics/btv761 (PMC4866520; doi:10.1093/bioinformatics/btv761)
Supplement: Supplementary Data [file supp_32_10_1441__index.html]

Vasohibins: new transglutaminase-like cysteine proteases possessing a non-canonical Cys-His-Ser catalytic triad — Vasohibins: new transglutaminase-like cysteine proteases possessing a non-canonical Cys-His-Ser catalytic triad — Supplementary Data 

# Vasohibins: new transglutaminase-like cysteine proteases possessing a non-canonical Cys-His-Ser catalytic triad

## Supplementary Data

files

- Supplementary Data - pdf file
