# Supplementary material for: Vasohibins: new transglutaminase-like cysteine proteases possessing a non-canonical Cys-His-Ser catalytic triad
Source: Bioinformatics. 2016 Jan 21;32(10):1441–5. doi: 10.1093/bioinformatics/btv761 (PMC4866520; doi:10.1093/bioinformatics/btv761)
Supplement: Supplementary Data [file supp_btv761_SanchezVASHSupplementary.pdf]

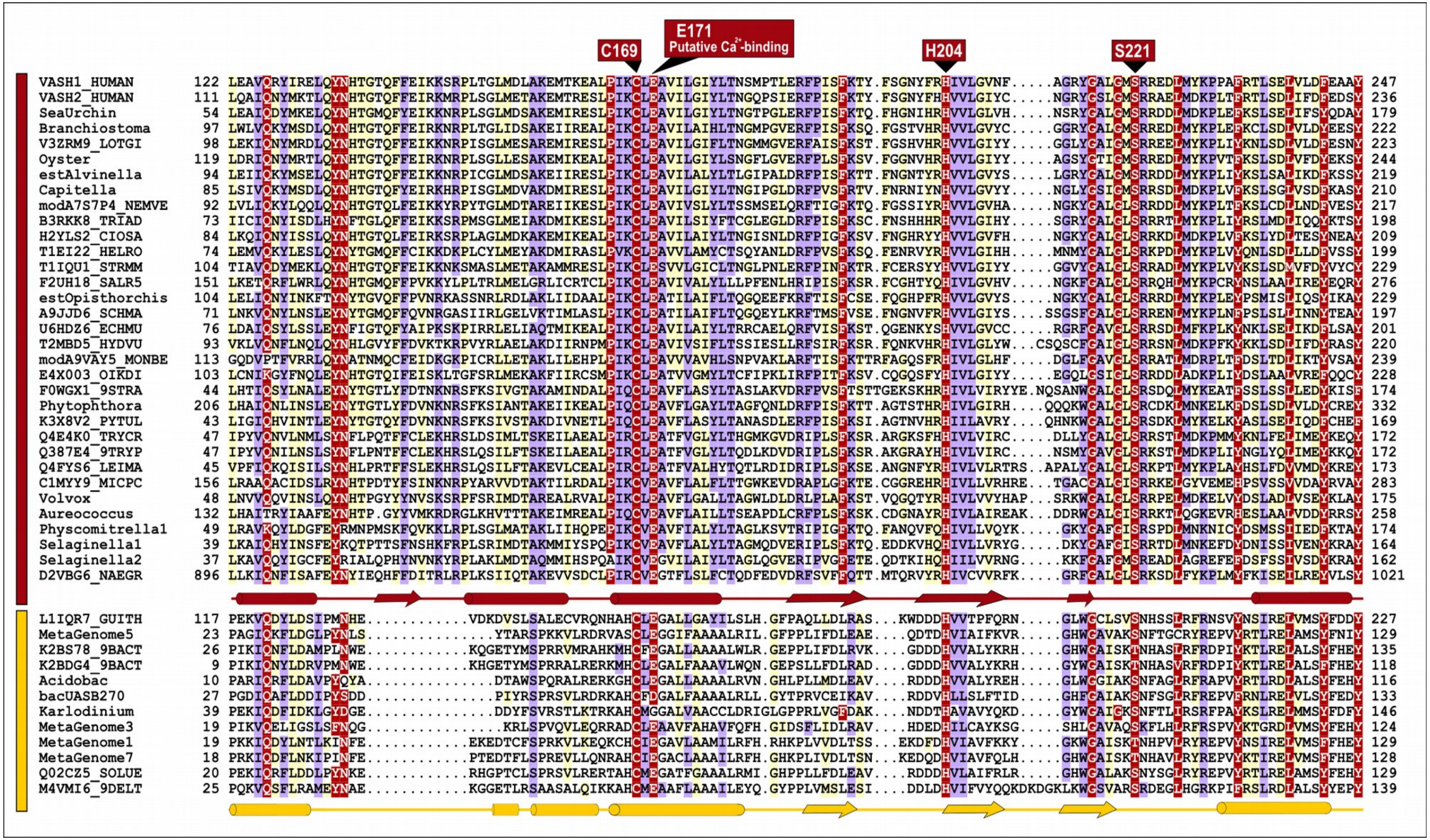
C169
E171
Putative Ca2+-binding
H204
S221
VASH1_HUMAN
VASH2_HUMAN
SeaUrchin
Branchiostoma
V3ZRM9_LOTGI
Oyster
estAlvinella
Capitella
modA7S7P4_NEMVE
B3RKK8_TRIAD
H2YLS2_CIOSA
T1EI22_HELRO
T1IQU1_STRMM
F2UH18_SALR5
estOpisthorchis
A9JJD6_SCHMA
U6HDZ6_ECHMU
T2MBD5_HYDVU
modA9VAY5_MONBE
E4X003_OIKDI
F0WGX1_9STRA
Phytophthora
K3X8V2_PYTUL
Q4E4K0_TRYCR
Q387E4_9TRYP
Q4FYS6_LEIMA
C1MYY9_MICPC
Volvox
Aureococcus
Physcomitrella1
Selaginella1
Selaginella2
D2VBG6_NAEGR
L1IQR7_GUITH
MetaGenome5
K2BS78_9BACT
K2BDG4_9BACT
Acidobac
bacUASB270
Karlodinium
MetaGenome3
MetaGenome1
MetaGenome7
Q02CZ5_SOLUE
M4VMI6_9DELT

Supplemental Fig. S1.

Representative multiple sequence alignment of the vasohibin and vasohibin-like families.

The alignment shows in Figure 1 corresponds to the currently defined core of the transglutaminase-like cysteine protease family (Ginalski *et al.*, 2004; Makarova *et al.*, 1999; Makarova *et al.*, 2000; Chatterjee *et al.* 2012), but we were able to extend the alignment between vasohibin and vasohibin-like families further toward their C-termini, including a predicted alpha-helix. Putative catalytic residues are shown in red. A putative calcium-binding residue E171 in human VASH1 is labelled. Families are indicated by coloured bars to the left of the alignment: vasohibin and vasohibin-like sequences are indicated in red and yellow, respectively. Secondary structure predictions (Jones, 1999) were performed independently for the vasohibin and vasohibin-like families. The limits of the protein sequences included in the alignment are indicated by flanking residue positions. Alignments were produced with T-Coffee (Notredame *et al.*, 2000) using default parameters and slightly refined manually. The alignment was presented with the program Belvu using a colouring scheme indicating the average BLOSUM62 scores (which are correlated with amino acid conservation) of each alignment column: red (>3), violet (between 3 and 1.5) and light yellow (between 1.5 and 0.5) (Sonnhammer and Hollich, 2005). Sequences were obtained from UniProt, GenBank and JGI (Joint Genome Institute) databases, but were supplemented by manually assembled ESTs and FGENESH+ predicted gene models. The "mod" prefix identifies UniProt sequences corrected by gene prediction software FGENESH+ (Solovyev *et al.*, 2006). The "est" prefix identifies consensus sequences manually reconstructed by assembling highly similar NCBI-GenBank expressed sequence tags from identical species (conceptual translations). Sequences are named according to their UniProt identification or common name. Database of origin, accession numbers, and species name: UniProt:VASH1_HUMAN, *Homo sapiens*; UniProt:VASH2_HUMAN, *Homo sapiens*; SeaUrchin, UniProt:UPI0000E46146, *Strongylocentrotus purpuratus*; Branchiostoma, JGI genome FGENESH+ predicted gene and NCBI_Est BW730620, *Branchiostoma floridae*; UniProt:V3ZRM9_LOTGI, *Lottia gigantea*; Oyster, UniProt:UPI0005C3CA01, *Crassostrea gigas*; estAlvinella, NCBI_Ests: GO188185 and GO140334, *Alvinella pompejana*; Capitella, JGI genome FGENESH+ predicted gene, *Capitella sp.*; UniProt:A7S7P4_NEMVE, *Nematostella vectensis*; UniProt:B3RKK8_TRIAD, *Trichoplax adhaerens*; UniProt:H2YLS2_CIOSA, *Ciona savignyi*; UniProt:T1EI22_HELRO, *Helobdella robusta*; UniProt:T1IQU1_STRMM, *Strigamia maritima*; UniProt:F2UH18_SALR5, *Salpingoeca rosetta*; estOpisthorchis, NCBI_Est:ES417530, *Opisthorchis viverrini*; UniProt:A9JJD6_SCHMA, *Schistosoma mansoni*; UniProt:U6HDZ6_ECHMU, *Echinococcus multilocularis*; UniProt:T2MBD5_HYDVU, *Hydra vulgaris*; UniProt:A9VAY5_MONBE, *Monosiga brevicollis*; UniProt:E4X003_OIKDI, *Oikopleura dioica*; UniProt:F0WGX1_9STRA, *Albugo laibachii*; Phytophthora, JGI genome FGENESH+ predicted gene, *Phytophthora ramorum*; UniProt:K3X8V2_PYTUL, *Pythium ultimum*; UniProt:Q4E4K0_TRYCR, *Trypanosoma cruzi*; UniProt:Q387E4_9TRYP, *Trypanosoma brucei brucei*; UniProt:Q4FYS6_LEIMA, *Leishmania major*; UniProt:C1MYY9_MICPC, *Micromonas pusilla*; Volvox, JGI genome FGENESH+ predicted gene, *Volvox carteri*; Aureococcus, JGI genome FGENESH+ predicted gene, *Aureococcus anophagefferens*; Physcomitrella1, JGI genome FGENESH+ predicted gene, *Physcomitrella patens*; Selaginella1, JGI genome FGENESH+ predicted gene, *Selaginella moellendorffii*; Selaginella2, JGI genome FGENESH+ predicted gene, *Selaginella moellendorffii*; UniProt:D2VBG6_NAEGR, *Naegleria gruberi*; UniProt:L1IQR7_GUITH, *Guillardia theta*; MetaGenome5, NCBI_gi:689677707, uncultured bacterium; UniProt:K2BS78_9BACT, uncultured bacterium; UniProt:K2BDG4_9BACT, uncultured bacterium; Acidobac, UniProt:UPI00036EC8CC, *Acidobacteriaceae bacterium*; bacUASB270, NCBI_gi:672404298, *bacterium UASB270*; Karlodinium, NCBI_Ests: EC161919 EC160142, *Karlodinium micrum*; MetaGenome3, NCBI_gi:735013117, *archaeon GW2011_AR4*; MetaGenome1, NCBI_gi:735021449, *archaeon GW2011_AR19*; MetaGenome7, NCBI_gi:735018696, *archaeon GW2011_AR17*; UniProt:Q02CZ5_SOLUE, *Solibacter usitatus*; UniProt:M4VMI6_9DELT, *Bdellovibrio exovorus*.

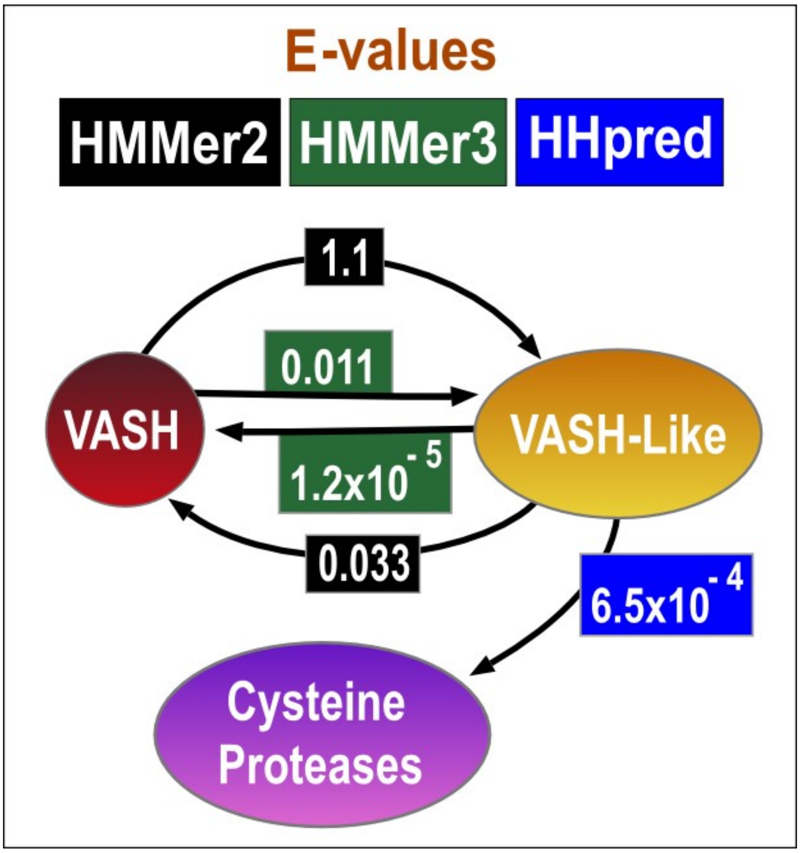

Supplemental Fig. S2.

HMMer and HHpred comparison E-values.

Numbers correspond to HMMer (versions 2 and 3) profile-versus-sequence (black and green boxes) and HHpred profile-versus-profile (blue box) comparison E-values from global profile search results (Eddy, 1996; Finn *et al.*, 2015; Soding *et al.*, 2005). Arrows indicate the profile search direction. Both versions of HMMer (used with default parameters) yielded highly concordant results. Profile-to-sequence (HMMer) and Profile-to-profile (HHpred) matches were evaluated in terms of an E-value, which is the expected number of non-homologous proteins with a score higher than that obtained for the database match. An E-value much lower than one indicates statistical significance.
